# Baseline plasma fibrinogen is associated with haemoglobin A1c and 2-year major adverse cardiovascular events following percutaneous coronary intervention in patients with acute coronary syndrome: a single-centre, prospective cohort study

**DOI:** 10.1186/s12933-019-0858-5

**Published:** 2019-04-23

**Authors:** Lisha Zhang, Chenbo Xu, Junhui Liu, Xiaofang Bai, Ruifeng Li, Lijun Wang, Juan Zhou, Yue Wu, Zuyi Yuan

**Affiliations:** 1 Department of Cardiovascular Medicine, The First Affiliated Hospital, Xi’an Jiaotong University, 277 West Yanta Rd., Xi’an, 710061 Shaanxi People’s Republic of China; 2 Department of Clinical Laboratory, The First Affiliated Hospital, Xi’an Jiaotong University, Xi’an, Shaanxi People’s Republic of China; 3 Key Laboratory of Molecular Cardiology, Shaanxi Province, Xi’an, Shaanxi People’s Republic of China; 4 Key Laboratory of Environment and Genes Related to Diseases (Xi’an Jiaotong University), Ministry of Education, Xi’an, Shaanxi People’s Republic of China

**Keywords:** Fibrinogen, Diabetes mellitus, HbA1c, Fasting blood glucose, Percutaneous coronary intervention, Acute coronary syndromes, Major adverse cardiovascular events

## Abstract

**Background:**

Despite revascularisation, a large proportion of acute coronary syndrome (ACS) patients continue to experience major adverse cardiovascular events (MACEs), which are worsened by diabetes mellitus (DM). Fibrinogen (FIB) is a risk factor for MACEs in coronary artery disease and often elevated in DM. However, the relationships between FIB, glucose metabolism (haemoglobin A1c [HbA1c] and fasting blood glucose [FBG]) and MACEs following percutaneous coronary intervention (PCI) in DM, non-DM or whole patients with ACS remains unknown.

**Methods:**

A total of 411 ACS patients undergoing PCI were enrolled in this study. We compared baseline FIB levels between DM (*n *= 103) and non-DM (*n *= 308) patients and divided participants into three groups according to FIB level, i.e. FIB-L, FIB-M and FIB-H, to compare baseline characteristics and MACEs. Linear regression analysis of the relationship between glucose metabolism and FIB, Cox regression, survival and landmark analyses of MACEs were also performed over a median of 27.55 months of follow-up.

**Results:**

Patients with DM had higher FIB levels than non-DM patients (3.56 ± 0.99 mg/dL vs. 3.34 ± 0.80 mg/dL, *P *< 0.05). HbA1c and FBG were significantly positively correlated with FIB in whole and DM patients but not in non-DM patients (all *P* < 0.05). Compared with the FIB-L group, the FIB-M (hazard ratio [HR] 1.797, 95% CI 1.117–2.892, *P* = 0.016) and FIB-H (HR 1.664, 95% CI 1.002–2.763, *P* = 0.049) groups were associated with higher MACEs in whole; the FIB-M (HR 7.783, 95% CI 1.012–59.854, *P* = 0.049) was associated with higher MACEs in DM patients. FIB was not associated with MACEs in non-DM patients. During landmark analysis, FIB showed better predictive value for MACEs after PCI in the first 30 months of follow up than in the subsequent period.

**Conclusion:**

In this study from China, FIB was positively associated with glucose metabolism (HbA1c and FBG) in whole and DM populations with ACS. Moreover, elevated baseline FIB levels may be an important and independent predictor of MACEs following PCI, especially amongst those with DM. However, as the follow-up period increased, the baseline FIB levels lost their ability to predict MACEs.

## Background

Coronary artery disease (CAD) is the leading cause of morbidity and mortality in developed countries. At present, the global burden of cardiovascular disease has shifted toward low- and middle-income countries (including China), wherein over 80% of global cardiovascular deaths occur [1, 2]. Revascularisation and antithrombotic strategies have achieved great success in reducing mortality from acute coronary syndromes (ACS), but the results remain unsatisfactory, especially amongst diabetic patients [3–7].

Fibrinogen (FIB) is an important part of the coagulation pathway and combines with receptors on the platelet membrane to form acute coronary thrombosis [8, 9]. As an acute phase reactant of inflammation, FIB is associated with long-term major adverse cardiovascular events (MACE) after percutaneous coronary intervention (PCI) [10, 11]. Previous studies show that FIB levels are higher in patients with diabetic mellitus (DM) than in controls, which may contribute to the higher thrombotic status of patients with the disease relative to those without [12, 13]. Amongst patients with acute myocardial infarction (AMI), admission haemoglobin A1c (HbA1c) is an important predictor of the severity of coronary artery stenosis in non-DM and DM patients [14]. However, few reports have investigated the relationship between baseline FIB levels, glucose metabolism (HbA1c and fasting blood glucose [FBG]) and MACEs in the ACS population after PCI with or without DM; of the studies available, very few originate in China.

The present study was performed to determine the relationship between baseline FIB levels and HbA1c or FBG and explore the role of baseline FIB levels with 2-year MACEs following PCI in ACS patients with or without DM in China.

## Materials and methods

### Study design and participants

This report presents a single-centre, prospective, observational, non-randomised and non-blind cohort study. Consecutive patients who were diagnosed with ACS and underwent PCI in the First Affiliated Hospital of Medical College of Xi’an Jiaotong University between January 2013 and February 2014 were enrolled in this investigation. The inclusion criteria were a diagnosis of ACS, including unstable angina (UA), non-ST-segment elevation myocardial infarction (NSTEMI) and STEMI. The exclusion criteria were as follows: severe renal and liver diseases, severe infections, immune system diseases, malignant tumours, blood system diseases, pregnancy, severe cerebrovascular diseases and prior history of surgical treatment within 2 weeks of this study.

ACS and DM were defined based on the criteria by the American Cardiology College and the American Diabetes Association [15, 16]. Composite end points, including all-cause death, non-fatal AMI, urgent coronary revascularisation, UA and cerebrovascular events (including cerebral bleeding or ischemic stroke), were defined as MACEs [17]. Patients underwent follow-up through personal or telephone interviews or reviews of medical records in our hospital until March 31, 2016; follow-up ended on the date of the first MACE occurrence. A total of 411 subjects who completed the follow-up were enrolled in the current study (median follow-up time, 27.55 months). Written informed consent was obtained from all study participants, and the study was approved by the ethics committee of the First Affiliated Hospital of Xi’an Jiaotong University (Ethical approval number: XJTU1AF2012LSK-312).

### Assessment of HbA1c and FIB

Peripheral blood samples were obtained from patients in a fasting state early in the morning after admission to the hospital prior to PCI. Baseline laboratory measurements, including complete blood count, creatinine, complete lipid panel, FIB, FBG and HbA1c, were performed at the biochemistry centre of our hospital by using standard biochemical techniques. The principle of Clauss coagulation was used to measure FIB levels: when the concentration of thrombin is high, the clotting time of the diluted plasma to be tested is inversely proportional to the level of FIB.

### Statistical analysis

Statistical analyses were performed by using SPSS 18.0 (SPSS Inc, Chicago, IL) and EmpowerStats (http://www.empowerstats.com/). Data are presented as frequencies and percentages for categorical variables and as mean ± SD for continuous variables. Variables of interest were compared using Student’s *t*-test, one-way ANOVA or the Chi squared test as appropriate. Linear regression analysis was used to calculate the correlation between HbA1c (or FBG) and FIB. We used Cox regression analysis to calculate HRs and 95% confidence intervals (CIs) for MACE comparisons. Landmark analyses (EmpowerStats) were performed according to a landmark point of the 30th month. HRs and 95% CIs were calculated separately for events occurring up to 30 months and those between the 31st month and the end of follow-up. A value of *P* < 0.05 was considered statistically significant.

## Results

### Basic characteristics of patients with and without DM

A total of 411 subjects who completed the follow-up were enrolled in the current study (median follow-up time, 27.55 months). The baseline characteristics of the DM, non-DM and whole patients are shown in Table 1.

**Table 1:** Basic characteristics for patients with DM, without DM and whole

Variable	Whole (n = 411)	Non-DM (n = 308)	DM (n = 103)	*P* value
FIB, mg/dL	3.39 ± 0.94	3.34 ± 0.99	3.56 ± 0.80	0.03
Age, year	60.6 ± 10.4	60.07 ± 10.38	62.41 ± 10.23	0.048
Male sex, %	77.1	79.2	70.9	0.081
BMI, kg/m^2^	24.78 ± 3.25	24.87 ± 3.32	24.65 ± 3.01	0.580
Past PCI or CABG, %	19.2	18.5	21.4	0.525
Past MI, %	15.8	15.3	17.5	0.594
Smoking, %	56.7	58.8	50.5	0.142
Hypertension, %	53.0	52.9	53.4	0.933
Family history, %	40.1	40.3	39.8	0.935
Ejection fraction, (%)	58.4 ± 12.3	58.73 ± 11.78	57.33 ± 13.80	0.371
HGB, g/L	139.3 ± 17.0	139.9 ± 18.0	137.84 ± 13.88	0.230
Platelet, 10^3^ cells/dL	191.30 ± 66.24	194.21 ± 67.85	186.10 ± 61.83	0.262
Creatinine, mg/dL	71.15 ± 33.76	71.28 ± 36.51	71.06 ± 22.51	0.942
HbA1c, %	6.43 ± 1.40	5.76 ± 0.38	8.36 ± 1.51	< 0.001
FBG, mmol/L	6.38 ± 2.43	5.55 ± 1.38	8.77 ± 3.16	< 0.001
LDL-C, mg/dL	2.22 ± 0.82	2.20 ± 0.82	2.28 ± 0.79	0.410
hsCRP, mg/dL	2.33 ± 2.30	2.22 ± 2.25	2.67 ± 2.42	0.181
CKMB, U/L	37.88 ± 555.93	37.12 ± 54.5	39.34 ± 58.94	0.739
Pro-BNP, pg/mL	837.36 ± 2076.66	805.19 ± 2097.0	970.62 ± 1960.9	0.474
Medication at discharge				
Aspirin, %	100	100	100	a
Clopidogrel	100	100	100	a
Statin, %	97.8	98.1	97.1	0.563
ACEI/ARB, %	92.5	92.5	92.2	0.921
CCB, %	24.3	24.4	24.3	0.974
β-blocker	88.6	89.0	87.4	0.662
ACS type				0.307
UA, n (%)	230 (55.9)	179 (58.1)	51 (49.5)	
STEMI, n (%)	122 (29.7)	86 (27.9)	36 (35.0)	
NSTEMI, n (%)	59 (14.4)	43 (14.0)	16 (15.5)	
MACE, %	39.2	41.6	32.0	0.087

Data are presented as mean ± SD or number (%)

*FIB* fibrinogen, *BMI* body mass index, *Past PCI or CABG* past percutaneous coronary intervention or coronary artery bypass grafting, *Past MI* past myocardial infarction, *HGB* hemoglobin, *HbA*_*1c*_ hemoglobin A1c, *FBG* fasting blood glucose, *CKMB* creatine kinase isoenzymes MB, *Pro-BNP* pro-B-type natriuretic peptide, *LDL-C* low-density lipoprotein cholesterol, *PLT platelets*, *ACEI* angiotensin-converting enzyme inhibition, *ARB* angiotensin receptor blocker, *CCB* calcium channel blocker, *ACS* acute coronary syndromes, *UA* unstable angina, *STEMI* ST-segment elevation myocardial infarction, *NSTEMI* non-ST-segment elevation myocardial infarction, *MACE* major adverse cardiovascular events

“a” represents *P* value = 1

### Comparison of clinical data between groups with different FIB levels

Patients were divided into three groups based on FIB level, and a comparison of the clinical data of these groups is shown in Table 2. The proportions of diabetics, baseline FBG and HbA1c levels, platelet count, hypersensitive C-reactive protein and pro-B-type natriuretic peptide increased as FIB level increased (all *P *< 0.05). Moreover, the higher the FIB level, the higher the proportion of patients with STEMI and the lower the proportion of patients with UA or NSTEMI (*P* = 0.045). The incidence of MACEs in the FIB-M (44.5%) and FIB-H (41.6%) groups was higher than that in the FIB-L (31.4%) group (*P *= 0.019); all-cause death showed the same result (FIB-L to FIB-M to FIB-H: 2.92% to 4.38% to 9.49%, *P *= 0.032). No significant difference in other risk factors and medication use at discharge was observed amongst the three groups.

**Table 2:** Basic characteristics for patients with different FIB levels

	Fibrinogen, mg/dL			*P* value
	FIB-L (1.76–2.91) N = 137	FIB-M (2.93–3.62) N = 137	FIB-H (3.63–8.14) N = 137	
FIB, mg/dL	2.53 ± 0.27	3.28 ± 0.20	4.21 ± 0.55	< 0.001
Age, year	60.0 ± 10.0	60.2 ± 10.0	61.7 ± 11.2	0.348
Male sex, %	75.2	81.0	75.2	0.414
BMI, kg/m^2^	24.83 ± 3.17	25.02 ± 3.17	24.4 ± 3.45	0.446
Past PCI or CABG, %	16.8	17.5	23.4	0.319
Past MI, %	13.9	14.6	19.0	0.456
Smoking, %	54.7	58.4	56.9	0.828
Diabetes, %	16.1	27.7	31.4	0.009
Hypertension, %	59.9	49.6	49.6	0.147
Family history, %	45.3	46.7	28.5	0.003
Ejection fraction, %	59.6 ± 11.3	57.1 ± 13.7	58.6 ± 11.7	0.247
HGB, g/L	139.6 ± 17.2	139.7 ± 17.6	138.5 ± 16.3	0.828
Platelet, 10^3^ cells/dL	181.92 ± 71.51	182.89 ± 51.07	210.04 ± 71.01	< 0.001
Creatinine, mg/dL	69.89 ± 24.97	68.74 ± 18.56	75.07 ± 50.39	0.271
HbA1c, %	6.08 ± 1.06	6.51 ± 1.50	6.72 ± 1.55	0.001
FBG, mmol/L	6.02 ± 2.28	6.36 ± 2.16	6.77 ± 2.80	0.043
LDL-C, mg/dL	2.27 ± 0.79	2.26 ± 0.78	2.14 ± 0.89	0.376
hsCRP, mg/dL	1.48 ± 1.78	2.19 ± 2.18	3.50 ± 2.51	< 0.001
CKMB, U/L	33.06 ± 48.26	38.48 ± 54.07	42.35 ± 64.79	0.809
Pro-BNP, pg/mL	473.09 ± 967.43	724.36 ± 1221.05	1343.61 ± 3244.03	< 0.001
Medication at discharge				
Aspirin, %	100	100	100	a
Clopidogrel, %	100	100	100	a
Statin, %	96.4	98.5	98.5	0.360
ACEI/ARB, %	94.9	94.2	88.3	0.078
CCB, %	26.3	19.0	27.7	0.278
ACS type				0.045
UA	58.4	56.9	52.3	
STEMI	21.9	29.9	37.2	
NonSTEMI	19.7	13.1	10.9	
MACE, %	31.4	44.5	41.6	0.019
All-cause death, %	2.92	4.38	9.49	0.032

Data are presented as mean ± SD or number (%)

*FIB* fibrinogen, *BMI* body mass index, *Past PCI or CABG* past percutaneous coronary intervention or coronary artery bypass grafting, *Past MI* past myocardial infarction, *HGB* hemoglobin, *HbA*_*1c*_ hemoglobin A1c, *FBG* fasting blood glucose, *hsCRP* hypersensitive C-reactive protein, *CKMB* creatine kinase isoenzymes MB, *Pro-BNP* pro-B-type natriuretic peptide, *LDL-C* low-density lipoprotein cholesterol, *PLT* platelets, *ACEI* angiotensin-converting enzyme inhibition, *ARB* angiotensin receptor blocker, *CCB* calcium channel blocker, *ACS* acute coronary syndromes, *UA* unstable angina, *STEMI* ST-segment elevation myocardial infarction, *NSTEMI* non-ST-segment elevation myocardial infarction, *MACE* major adverse cardiovascular events

“a” represents *P* value = 1

### Association between HbA1c/FBG and FIB

**Fig. 1:**
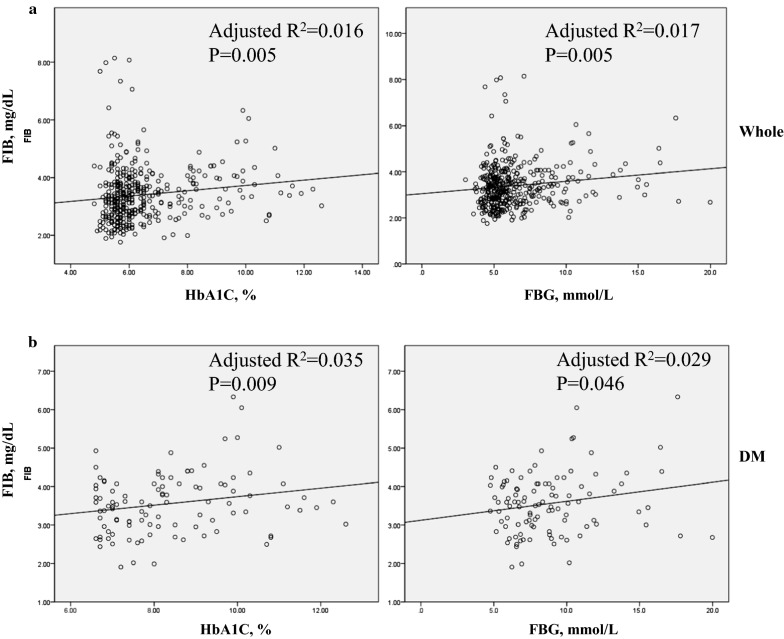
Linear regression analysis of the relationship between glucose metabolism and FIB. **a** Linear regression analysis of the relationship between glucose metabolism (HbA1c and FBG) and FIB in whole patients with ACS after PCI. **b** Linear regression analysis of the relationship between glucose metabolism (HbA1c and FBG) and FIB in ACS patients with DM after PCI. *FIB* fibrinogen, *DM* diabetes mellitus, *HbA1c* Haemoglobin A1c, *FBG* fasting blood glucose

To investigate the relationship between glucose metabolism indices and FIB, we utilised linear regression analysis (Table 3). Admission HbA1c (*R*^*2*^ = 0.016; 95% CI 0.027–0.158, *P* = 0.005) and FBG level (*R*^*2*^ = 0.017; 95% CI 0.016–0.092, *P* = 0.005) were significantly positively correlated with FIB in whole ACS patients. In ACS patients with DM, admission HbA1c (*R*^*2*^ = 0.035; 95% CI 0.009–0.212, *P* = 0.009) and FBG level (*R*^*2*^ = 0.029; 95% CI 0.001–0.098, *P* = 0.046) were also positively correlated with FIB. However, in the non-DM population, no significant relation was found between FIB and HbA1c or FBG (*P* > 0.05). The corresponding graphs are shown in Fig. 1.

**Table 3:** Linear regression analysis between glucose metabolism and FIB in patients with DM, without DM and whole

Variable	Adjusted R square	Coefficient	95% CI	SEM	*P* value
Whole					
HbA1C, %	0.016	0.137	0.027 to 0.158	0.939	0.005
FBG, mmol/L	0.017	0.138	0.016 to 0.092	0.939	0.005
DM					
HbA1C, %	0.035	0.210	0.009 to 0.212	0.781	0.009
FBG, mmol/L	0.029	0.197	0.001 to 0.098	0.784	0.046
Non-DM					
HbA1c, %	− 0.001	0.052	− 0.157 to 0.423	0.989	0.367
FBG, mmol/L	0.001	0.063	− 0.035 to 0.126	0.988	0.267

*FIB* fibrinogen, *DM* diabetes mellitus, *HbA1c* Hemoglobin A1c, *FBG* fasting blood glucose, *CI* confidence interval, *SEM* standard error of measurement

### Cox regression and landmark analysis in patients with ACS

**Fig. 2:**
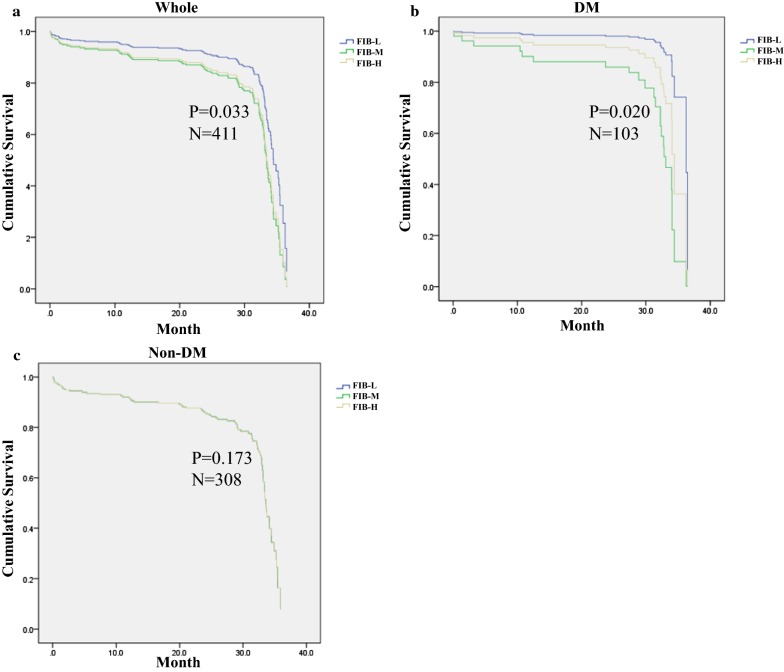
Kaplan–Meier survival curves for freedom from MACEs in the whole, non-DM and DM patient groups. Kaplan–Meier survival curves for freedom from MACEs in **a** the whole population by FIB level, **b** DM by FIB level and **c** non-DM by FIB level. *FIB* fibrinogen, *DM* diabetes mellitus

Cox regression analysis revealed that, when compared with the FIB-L group, the FIB-M (HR 1.797, 95% CI 1.117–2.892, *P *= 0.016) and FIB-H (HR 1.664, 95% CI 1.002–2.763, *P *= 0.049) groups were associated with increased MACEs over a median of 27.55 months of follow-up in the whole population; and FIB-M (HR 7.783, 95% CI 1.012–59.854, *P *= 0.049), but not HbA1c and FBG, was associated with increased MACEs in DM patients. In addition, baseline FIB levels revealed no relationship with MACEs in non-DM patients (Table 4). The corresponding Kaplan–Meier curves are shown in Fig. 2.

**Table 4:** Cox regression analysis of MACE in patients with DM, without DM and whole

Variable	Hazard Ratio	95% CI	SEM	*P* value
Whole				
Hypertension	1.044	1.023 to 1.066	0.010	< 0.001
CKMB	0.993	0.989 to 0.997	0.002	0.002
FIB-L				0.033
FIB-M^a^	1.797	1.117 to 2.892	0.243	0.016
FIB-H^a^	1.664	1.002 to 2.763	0.259	0.049
DM				
Hypertension	1.033	1.005 to 1.061	0.014	0.021
FIB-L				0.020
FIB-M^a^	7.783	1.012 to 59.854	1.041	0.049
FIB-H^a^	3.398	0.407 to 28.347	1.082	0.258
Non-DM				
Hypertension	1.055	1.028 to 1.083	0.013	< 0.001
FIB-L				0.173
FIB-M^a^	1.797	0.948 to 3.408	0.327	0.895
FIB-H^a^	1.926	0.988 to 4.042	0.359	0.094

*MACE* major adverse cardiovascular events, *FIB* fibrinogen, *CKMB* creatine kinase isoenzymes MB, *DM* diabetes mellitus, *CI* confidence interval, *SEM* standard error of measurement

^a^Compared with FIB-L group

**Fig. 3:**
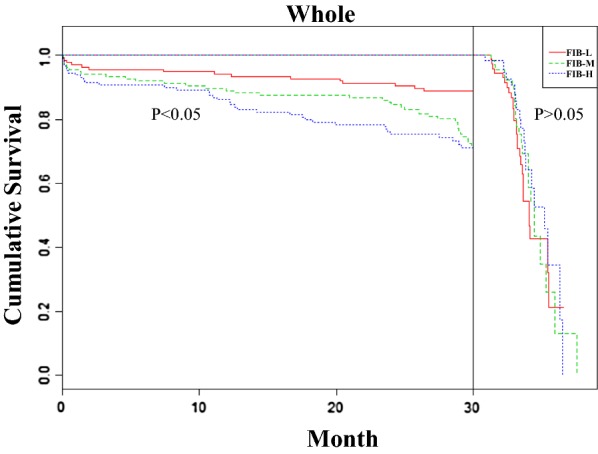
Landmark analysis of MACEs in ACS patients after PCI. Kaplan–Meier survival curves (divided into two parts by the landmark point of 30 months) for freedom from MACEs in the whole population by FIB level. *ACS* acute coronary syndrome, *FIB* fibrinogen, *DM* diabetes mellitus, *PCI* percutaneous coronary intervention, *HR* hazard ratio, *CI* confidence interval

Because the number of patients with DM in this work was not adequate for landmark analysis, the overall population was subjected to landmark survival analysis with a landmark point of 30 months. The results in Table 5 show significantly more 2-year MACEs in the FIB-M (HR 3.798, 95% CI 1.508–9.564, *P *= 0.005) and FIB-H (HR 4.405, 95% CI 1.587–12.227, *P *= 0.004) groups than in the FIB-L group within 30 months of follow up (MACEs [FIB-L to FIB-M to FIB-H]: 10.9% to 26.3% to 27.7%) but not in the later period (between the 31st month and end of follow-up) in the whole population (MACEs [FIB-L to FIB-M to FIB-H]: 36% to 34% to 30.6%). Significant interactions were observed between time and FIB with respect to MACEs. The corresponding Kaplan–Meier curves are shown in Fig. 3.

**Table 5:** Landmark analysis of MACE in ACS patients after PCI

Variable	MACE, %	Hazard ratio	95% CI	SEM	*P* value
≤ 30 months					
Hypertension		1.047	1.014 to 1.080	0.016	0.005
PLT		1.006	1.001 to 1.011	0.003	0.012
FIB-L	10.9				
FIB-M^a^	26.3	3.798	1.508 to 9.564	0.471	0.005
FIB-H^a^	27.7	4.405	1.587 to 12.227	0.521	0.004
31 months to maximum follow-up					
Hypertension		1.002	1.001 to 1.003	0.001	< 0.001
Smoking		1.039	1.004 to 1.076	0.018	0.029
CRE		0.973	0.949 to 0.997	0.013	0.030
FIB-L	36				
FIB-M^a^	34	1.458	0.548 to 3.876	0.499	0.450
FIB-H^a^	30.6	1.356	0.395 to 4.656	0.629	0.628

*ACS* acute coronary syndrome, *PCI* percutaneous coronary intervention, *MACE* major adverse cardiovascular events, *PLT* platelet, *FIB* fibrinogen, *CRE* creatinine, *CI* confidence interval, *SEM* standard error of measurement

^a^Compared with FIB-L group

## Discussions

In the current study, we noted that FIB levels were higher in Chinese ACS patients with DM than in non-DM patients after PCI. Baseline plasma FIB was related to HbA1c and FBG, and Cox regression analysis demonstrated that elevated baseline FIB levels are an important and independent predictor of MACEs in the whole and DM patient populations with ACS after PCI but not in non-DM patients over a median of 27.55 months of follow-up. Moreover, landmark survival analysis indicated that, over the first 30 months, patients with elevated FIB showed a large number of MACEs. From the 31st month to the end of follow-up, no significant association was found between FIB level and MACEs; this finding suggests that the relationship between FIB and MACEs is more reliable over short follow-up times than over longer periods. To the best of our knowledge, this study is first to investigate the relationship between FIB, glucose metabolism (HbA1c and FBG) and 2-year MACEs in ACS patients undergoing PCI with or without DM in China.

Fibrinogen is converted into insoluble fibrin by thrombin and expose polymerisation sites facilitating clot formation during activation of the coagulation cascade [18, 19]. Individuals suffering from diabetes exhibit higher levels of plasma FIB than those without type-2 diabetes mellitus (T_2_DM) [20, 21]. Inflammation is a common antecedent of atherosclerosis and diabetes, and FIB plays essential roles in inflammation and tissue repair [22, 23]. FIB is related to insulin sensitivity and insulin resistance causes oxidative stress via thrombin formation and the following FIB synthesis, which promotes diabetes complications and adverse clinical consequences [24–28]. However, whether HbA1c and FBG levels are correlated with FIB in patients with ACS, DM-ACS and non-DM-ACS undergoing PCI remains unclear. HbA1c, which reflects the 3-month average plasma glucose concentration, is a marker of long-term glucose management, and HbA1c levels are associated with the prognosis of AMI [29, 30]. In the present study, the mean FIB level of DM patients was higher than that of non-DM patients. In addition, linear regression analysis showed that admission HbA1c and FBG were positively correlated with FIB in patients with ACS and DM-ACS but not in patients without DM. Our study not only confirms the relationship between FIB and diabetes but also further assesses the relationship between FIB and glucose metabolism indicators in ACS patients with or without DM. However, conflicting results were found. The prospective Multi-Ethnic Study of Atherosclerosis showed that, after adjustment, FIB levels are no longer related to the onset of diabetes in the entire sample [31]. Similarly, the multicentre epidemiological Insulin Resistance Atherosclerosis Study showed that changes in FIB are not substantially related to incident diabetes [32]. Obviously, the target population of these two studies are participants without CAD or DM, and their aim is to explore the association between FIB and incident diabetes; these details are very different from those of the current study.

Patients with DM often have accelerated atherosclerosis and more serious clinical outcomes than those without the disease [33]. A nationwide study from Korea that used data from four consecutive nationwide databases revealed that the absolute burden of CAD remains high as the number of people with diabetes continues to increase [34]. Previous studies have reported relations between elevated FIB levels and adverse cardiovascular events in CAD and DM patients [10, 11, 35–38]. FIB is regarded as a risk factor in prognostic models for patients with T_2_DM, but few studies have explored these relations in patients with DM-ACS, non-DM ACS and whole who underwent PCI [39]. The major outcome of this study shows that FIB level is an important and independent predictor of 2-year MACEs in patients in the whole ACS and ACS combined with DM populations. By contrast, in non-DM patients, FIB level reflected no relationship with MACEs. As described above, FIB is positively correlated with HbA1c and FBG. In addition, elevated baseline FIB may potentiate MACE through platelet crosslinking, clot formation and arterial thrombosis [35]. These findings may explain why FIB exhibits no predictive value for MACEs in ACS patients without DM in this study. In the ADVANCE study, a case-cohort study including 3865 patients with T_2_DM and baseline CAD or risk factors, IL-6 levels, but not CRP or FIB levels, were significant to the prediction of macro-vascular events and mortality [40]. CAD is a clinical syndrome with high heterogeneity and different disease severities and prognoses. Different types of CAD may lead to differences in the research results and differences in time during which the studies were conducted may also contribute to the inconsistent results.

Many clinical studies have shown that the risks of target lesion failure, safety and efficacy outcomes amongst patients undergoing PCI are similar after implantation of third-generation drug eluting stents with biodegradable polymers or second-generation drug eluting stents with durable polymers [41–43]. In addition, patients with and without DM show different performance in terms of safety and effectiveness for the same kind of stent, thus suggesting the existence of differences between groups with and without diabetes [44, 45]. The stents implanted in all patients of the present study were second-generation drug-eluting stents; as such, the effect of different types of implanted stents on cardiovascular events was minimised.

Table 5 shows the results of landmark analyses of the MACEs. FIB consistently revealed better predictive value for MACEs after PCI in the first 30 months than in the subsequent period (between the 31st month and end of follow-up). Significant interactions were found between time and FIB with respect to MACEs, which suggests that baseline FIB levels cannot accurately reflect patients’ fibrinolysis and coagulation status with prolonged follow-up. Additional studies addressing the effects of FIB level variability over time or the role of FIB isoform variability are required.

This study presents a number of advantages. First, we explored the relationship between FIB and glucose metabolism (HbA1c and FBG) in the whole, DM and non-DM populations with ACS. Results showed that baseline FIB is positively associated with HbA1c and FBG in the whole and DM populations with ACS, which helps enhance our understanding of the relationship between diabetes and FIB. Secondly, several reports on the relationship between FIB and cardiovascular events in patients with diabetes and/or CAD have been published [38, 39]. Some studies focus on the association between FIB and long- or short-term MACEs in patients with ACS [10, 11, 35]. However, few researchers have assessed 2-year MACEs in ACS populations after PCI with or without DM. Despite revascularisation, a large proportion of ACS patients continue to experience MACEs, which is worsened by DM. Thus, determining the relationship between FIB and MACEs in ACS patients with DM and without DM is meaningful. Finally, few scholars have explored the relationship between FIB and MACEs in a Chinese ACS population after PCI. In a study from Beijing, the association between FIB and cardiovascular events in patients with stable angina pectoris and T_2_DM was assessed [38]. However, the target population of this work is very different from that in the present study. Another study showed that FIB level at admission is independently associated with death risk amongst Chinese patients with ACS [46]. However, this work did not explore the relationship between FIB and glucose metabolism or the relationship between FIB and 2-year MACEs in ACS patients after PCI with and without DM. Thus far, the present work is the first to study the relationship between FIB, glucose metabolism (HbA1c and FBG) and 2-year MACEs after PCI in ACS patents with and without DM in a prospective cohort from China.

This study features some limitations. Firstly, this study is a single-centre observational cohort study, which is less convincing than clinical trial studies. Secondly, only 411 ACS patients were enrolled in this work, amongst which only 103 patients had T_2_DM; this limitation may affect the reliability of the results. Finally, the study only included ACS patients undergoing PCI, which suggests that the study results may not be generalisable to all ACS patients.

## Conclusions

Elevated baseline plasma FIB levels were positively correlated with HbA1c and FBG and associated with 2-year MACEs (median follow-up, 27.55 months) independent of HbA1c and inflammatory status in whole ACS and DM-ACS patients following PCI but not in ACS patients without DM. However, as the follow-up period increased, baseline FIB levels lost their ability to predict MACEs.

## Abbreviations

FIB: fibrinogen
HbA1c: hemoglobin A1c
FBG: fasting blood glucose
AMI: acute myocardial infarction
T_2_DM: type 2 diabetes mellitus
BMI: body mass index
HGB: hemoglobin
hsCRP: hypersensitive C-reactive protein
CKMB: creatine kinase isoenzymes MB
Pro-BNP: pro-B-type natriuretic peptide
LDL-C: low-density lipoprotein cholesterol
PLT: platelets
ACEI: angiotensin-converting enzyme inhibition
ARB: angiotensin receptor blocker
CCB: calcium channel blocker
CAD: coronary artery disease
ACS: acute coronary syndromes
UA: unstable angina
STEMI: ST-segment elevation myocardial infarction
NSTEMI: non-ST-segment elevation myocardial infarction
MACE: major adverse cardiovascular events
PCI: percutaneous coronary intervention
CABG: percutaneous coronary intervention or coronary artery bypass grafting
